# Photon-counting CT iodine maps for late iodine enhancement: a retrospective feasibility study of image quality and reader confidence

**DOI:** 10.1186/s41747-026-00774-3

**Published:** 2026-08-10

**Authors:** Viktor Hartung, Philipp Gruschwitz, Julia Karin Serfling, Jaykumar Padodara, Ali Dayoub, Ramin Dazeh, Linus Desing, Alena Kollmann, Nils Petri, Jan-Peter Grunz, Henner Huflage

**Affiliations:** 1 Department of Diagnostic and Interventional Radiology, University Hospital Würzburg, Würzburg, Germany; 2 Department of Internal Medicine I, University Hospital Würzburg, Würzburg, Germany

**Keywords:** Contrast media, Coronary artery disease, Iodine, Myocardium, Tomography (X-ray computed)

## Abstract

**Objective:**

Myocardial scar assessment is clinically relevant in advanced coronary artery disease, but late iodine enhancement (LIE) CT remains limited by image contrast and validation requirements. This study aimed to evaluate quantitative image quality and reader confidence of photon-counting detector computed tomography (PCD-CT)-derived iodine maps for ischemic-type LIE after coronary computed tomography angiography (CTA).

**Materials and methods:**

This is a retrospective single-center study including patients referred for work-up of coronary artery disease. LIE was acquired 5 min after contrast injection for coronary CTA (1.5 mL/kg bodyweight, 350 mg/mL iodine concentration). Iodine maps were reconstructed (Qr32, 4-mm slices). Contrast-to-noise ratios (CNR) were measured, including scar–myocardium CNR in patients with LIE. Eight readers rated image-quality-based confidence for assessing ischemic-type LIE at ≤ 25%, ≤ 50%, and > 50% transmural extent. Inter-reader agreement was assessed using ICC.

**Results:**

Twenty-nine patients (13 women, age 73 ± 9 years, BMI 26.7 ± 6.6 kg/m^2^) were analyzed; LIE was present in 19/29 patients. Mean scar–myocardium contrast was 26.5 ± 9.8 HU. Median confidence increased with transmural extent: moderate for < 25% myocardium thickness (median 3, IQR 3-4), good for ≤ 50% (median 2, IQR 1–3) and high for > 50% (median 1, IQR 1–2, all *p *< 0.001). Inter-reader agreement was good across transmurality categories (ICC > 0.795, *p* < 0.001).

**Conclusion:**

PCD-CT iodine maps acquired 5 min after coronary CTA provided measurable scar conspicuity and high reader confidence for transmural LIE exceeding 50% wall thickness in this small, selected feasibility cohort. Diagnostic accuracy and clinical impact require prospective validation against LGE-MRI.

**Key Points:**

- **Question:** Delayed PCD-CT iodine maps acquired 5 min after coronary CTA provided interpretable late iodine enhancement images in selected patients.
- **Findings:** In 29 patients, scar–myocardium CNR was 16.2 ± 7.3; reader confidence was high for enhancement involving 50% of myocardial wall thickness and lower for ≤ 25% involvement.
- **Relevance statement:** These feasibility data support prospective validation of delayed iodine maps as a targeted adjunct when myocardial scar information is clinically relevant during cardiac CT work-up.

**Graphical Abstract:**

**Figure:**
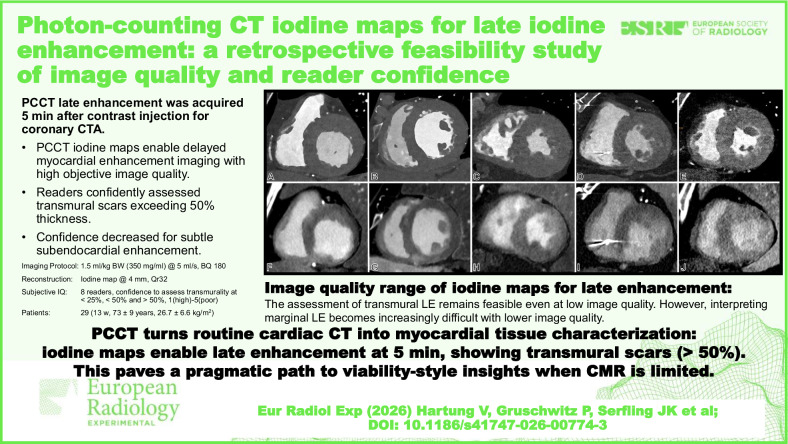

## Introduction

Cardiovascular disease remains the primary cause of morbidity and mortality globally [1]. Computed tomography angiography (CTA) of the coronaries has long become a mainstay in acute and chronic coronary artery disease [2, 3]. Information on myocardial scar burden and transmurality may be clinically relevant, particularly when revascularization is being considered [4–6].

Late gadolinium enhancement (LGE) MRI remains the reference standard for myocardial scar assessment [7] with significant influence on patient outcomes [8]. Unsurprisingly, demand has multiplied in recent years [9], largely due to demographic effects and technical advances. It is estimated that up to 15–30% of patients eligible or referred for cardiac MR might have contraindications, such as non-MRI conditional devices [10], renal impairment [11], claustrophobia or other factors (*i.e.*, severe obesity, inability to lie flat, severe arrhythmias, etc.). Furthermore, system-level barriers to CMR access, such as capacity issues, perceived high costs or lack of specialized expertise might hinder an additional 10–25% of patients from receiving a scan [12].

Reports of myocardial viability assessment using CT-derived late iodine enhancement (LIE) have been around for more than three decades [13], and the first experiments to integrate LIE into the workup of myocardial infarction are at least 20 years old [14, 15]. While LIE has been purported as an acceptable alternative to MRI-based diagnostics [16] with high accuracy in comparison to LGE [17], LIE has not been incorporated into any major imaging guidelines as of this writing. Reasons include low contrast-to-noise ratio (CNR) and reduced diagnostic confidence when using conventional CT scanners, as well as the markedly increased contrast agent volume needed for diagnostic imaging.

Photon-counting detector CT provides energy-resolved data with high dose efficiency and enables iodine map reconstructions from cardiac CT datasets even for ECG-gated, high-pitch acquisition scans [18, 19]. However, despite their advantages in the context of pulmonary embolism detection [20], photon-counting detector computed tomography (PCD-CT)-derived iodine maps have not been thoroughly investigated for the analysis of LIE in cardiac CT imaging thus far [21]. These technical features may be advantageous for delayed iodine imaging, particularly when iodine maps are acquired after coronary CTA without an additional contrast bolus. However, before broader clinical validation can be pursued, the feasibility, image quality, and reader confidence of this approach need to be characterized.

Consequently, the purpose of this study was to evaluate the technical feasibility, quantitative image quality, and multi-reader confidence of PCD-CT-derived iodine maps for assessing ischemic-type LIE after coronary CTA in a selected clinical cohort. We hypothesized that delayed iodine maps would provide sufficient scar–myocardium contrast and reader confidence for transmural LIE. Diagnostic accuracy against LGE-MRI was outside the scope of this feasibility study.

## Methods

This retrospective, single-center, observational feasibility study analyzed image quality and reader confidence in PCD-CT LIE.

### Patients

Patients scanned at our academic university hospital between June and August 2024 were retrospectively analyzed. Age of 18 years or older was mandatory for study inclusion. Patients were referred for a CT work-up of known or suspected coronary artery disease. In our institutional workflow, delayed LIE imaging was not performed as a general add-on to all coronary CTA examinations. It was reserved for selected patients in whom myocardial scar information was considered clinically relevant during CT work-up, including patients with known or suspected advanced coronary artery disease, prior myocardial infarction, myocardial thinning or wall-motion abnormalities on available imaging or planned revascularization. In patients referred for suspected coronary artery disease, the delayed acquisition was omitted when coronary CTA showed no relevant coronary artery disease at the scanner console. LGE-MRI remained the reference standard for myocardial viability assessment. However, CT-based LIE was used selectively when additional scar information during the CT examination was considered clinically useful, for example, because MRI was unavailable, contraindicated, previously performed, or not expected to be obtained in a clinically useful time frame. This workflow enriched the cohort for coronary artery disease and may limit generalizability to unselected populations. For patients who did receive the delayed scan, exclusion criteria were deviations from the standard protocol with potential impact on LIE, excessive artefacts, and premature abortion of the scan.

### Ethics declaration

The institutional review board approved this retrospective analysis and waived the requirement for written informed consent (protocol no. 20241021 01).

### Imaging protocol and reconstruction parameters

LIE scans were acquired 5min after iodine contrast injection, used for coronary CTA, using a first-generation dual-source PCD-CT system (Naeotom Alpha, software version VA60, Siemens Healthineers, Forchheim, Germany) in standard-resolution mode, *i.e*., employing 2 × 2 pixel binning for a physical pixel size of 0.275 × 0.322 mm. Detector collimation was 144 × 0.4 mm with a z-coverage of 57.6 mm. High-pitch spiral CT scans (pitch 3.2) were performed with prospective ECG-triggering at a 250 ms R-R interval. Automatic tube current modulation was employed (CARE Dose4D, Siemens Healthineers) with automatic exposure control (IQ level 180). Patients received 1.5 mL iodine contrast agent per kilogram bodyweight up to a maximum of 100 mL iomeprol with 350 mg/mL iodine concentration (Imeron 350, Bracco Imaging, Konstanz, Germany) at a flow rate of 5 mL/s followed by a 20 mL saline chaser.

**Fig. 1:**
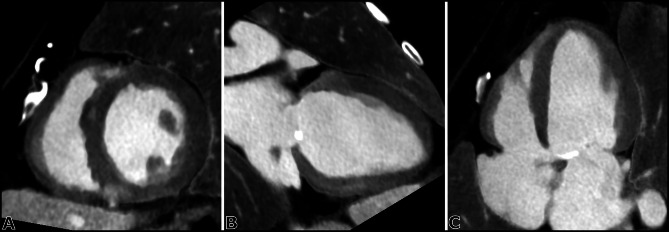
Late iodine enhancement reconstructions. Images from a 62-year-old man with clinically suspected coronary artery disease and cardiovascular risk factors. Late iodine enhancement at the mid-ventricular short axis (**A**), two-chamber view (**B**), and four-chamber view (**C**). No ischemic-type myocardial scar or fibrosis was found. Notice the hinge point late enhancement in (**A**) as a commonly encountered but non-specific finding of remodeling

The delayed iodine map acquisition was performed without additional contrast; the coronary CTA dataset served as an anatomical reference. PCD-CT datasets were used to reconstruct iodine distribution maps with 4 mm slice thickness in four chamber, two chamber, and short axis orientations (Fig. 1) employing a quantitative soft tissue kernel (Qr32). Iterative reconstruction strength was set to the highest applicable level (QIR level 4, Siemens Healthineers) to minimize image noise. Field-of-views ranged from 180–200 mm, and the image matrix was 512 × 512 pixels in all studies (Table 1). For the computation of CNR, iodine maps were calculated in Hounsfield units rather than iodine concentration, whereas 1 HU ~ 38 μg/cm^3^ [22].

**Table 1:** Scan and reconstruction parameters

Scan parameters	
Contrast agent volume (mL/kg)	1.5 mL/kg bodyweight, 100 mL max.
Detector collimation (mm)	144 × 0.4
Rotation time (s)	0.25
Pitch	3.2
Peak tube voltage (kVp)	140
IQ index (dimensionless)	180
ECG triggering	250 ms R-R-interval
Reconstruction parameters	
Field of view	Variable (180–200 mm)
Matrix	512 × 512
Spectral information	Iodine maps
Reconstruction kernel	Qr32
Iterative reconstruction	QIR (quantum iterative reconstruction) level 4
Slice thickness (mm)	4
Multi-planar reconstruction	4-chamber, 2-chamber, short axis

Continuous variables are given as mean ± standard deviation

### Contrast-to-noise ratio

**Fig. 2:**
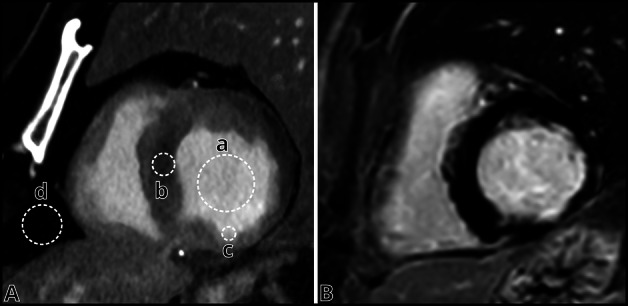
Region of interest measurements. Images from a 71-year-old man with a history of coronary artery disease. Mid-ventricular short axis late iodine enhancement (**A**) with corresponding late gadolinium enhancement from a previous cardiac MRI scan (**B**). Circular regions of interest indicate measurements for CT attenuation of the blood pool (HU_blood_, **a**), myocardium (HU_myocardium_, **b**) and late enhancing scar (HU_scar_, **c**), as well as image noise measured as the standard deviation of CT attenuation in fat (SD_fat_, **d**)

Representative circular regions of interest (> 30 mm^2^) were drawn by a board-certified radiologist with 8 years of training in the field (Fig. 2). The standard deviation of the attenuation in unaltered fat tissue was used as a surrogate of image noise (SD_fat_). Extracardiac fat was chosen because it provides a relatively homogeneous region outside the enhancing myocardium and blood pool, thereby avoiding direct influence of myocardial texture, focal scar enhancement or intravascular iodine heterogeneity on noise estimation. Furthermore, the CT attenuation in the mid-myocardial septum (HU_myocardium_) and blood pool (HU_blood_) was measured. In cases of LIE, CT attenuation within the scar tissue was recorded (HU_scar_). Based on these measurements, CNR was calculated as follows:

$${{CNR}}_{{myocardium}}=\left({{HU}}_{{blood}}-{{HU}}_{{myocardium}}\right)/{{SD}}_{{fat}}{{\rm{and}}}$$

$${{CNR}}_{{scar}}=\left({{HU}}_{{scar}}-{{HU}}_{{myocardium}}\right)/{{SD}}_{{fat}}.$$

CNR_myocardium_ reflects blood–myocardium separability; CNR_scar_ reflects scar conspicuity against myocardium.

### Diagnostic confidence

**Fig. 3:**
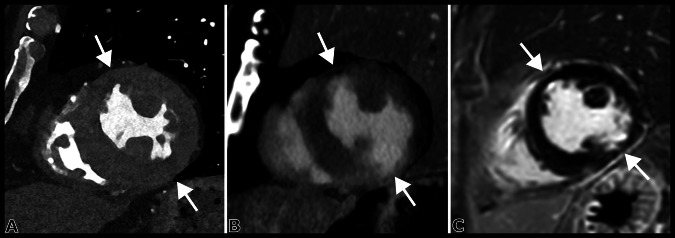
Myocardial viability. Images from a 67-year-old woman with suspected progression of known coronary artery disease. Mid-ventricular short axis from coronary CT angiography (**A**), late iodine enhancement maps (**B**), and late gadolinium enhancement from an MRI scan acquired 2 years before (**C**). The MRI image is shown for anatomical illustration only and was not used as a reference standard in the present analysis. Arrows indicate regions of ischemic scarring. Notice the relative myocardial thinning discernible in **A**, and the late enhancement in **B** exceeding 50% of the myocardium thickness, which corresponds well with the late gadolinium enhancement in **C**. The CT scan revealed no signs of coronary artery disease progression

Eight radiologists with 1–8 years of training in cardiac imaging, including LIE interpretation, rated their confidence in interpreting ischemic type subendocardial LIE involving up to 25%, up to 50%, or more than 50% of myocardial wall thickness on a five-point rating scale (1 =  high confidence, 2 =  good confidence, 3 = moderate confidence, 4 = fair confidence, 5 = poor confidence), based on overall map interpretability (*i.e*., whether enhancement of the stated transmural extent would be discernible). Readers received written definitions of the three transmurality categories and reviewed cases in random order. No clinical history and no prior reports were provided. The accompanying coronary CTA scans were provided to assist in the accurate delineation of myocardial borders (Fig. 3).

### Statistical analysis

Statistical analysis was performed using Prism 10 (GraphPad Software Inc., Boston, Massachusetts, USA). Statistical significance is indicated by an alpha level < 0.05. Continuous variables are given as mean ± standard deviation, and ordinal data as median values with interquartile ranges. Parametric imaging parameters were analyzed using one-way analyses of variance, and *p*-values of pairwise post-hoc tests were corrected for multiple comparisons applying the Tukey method. Given the retrospective feasibility design and small sample size, quantitative comparisons were considered exploratory. No formal comparison with MRI, conventional delayed enhancement CT, energy-integrating detector CT, or dual-energy CT was performed; therefore, no claims of diagnostic accuracy or technical superiority over these modalities were made. To assess interrater agreement, the intraclass correlation coefficient (ICC) was calculated based on absolute agreement in a two-way random effects model. Agreement was interpreted following established guidelines, whereas an ICC < 0.5 indicated poor agreement, an ICC of 0.5–0.75 indicated moderate agreement, ICC 0.75–0.9 indicated good agreement and ICC > 0.9 indicated excellent agreement [23].

## Results

### Patient characteristics

**Fig. 4:**
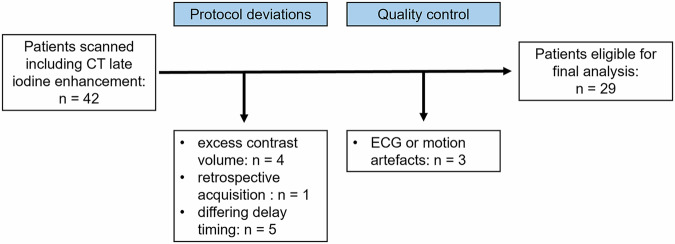
Patient inclusion flowchart

Following the inclusion criteria, 42 patients received an LIE scan. Of these, 13 had to be excluded for protocol deviations with potential impact on quantitative image parameters of LIE (*N* = 10) or quality issues not related to LIE, such as significant ECG- or motion artifacts (*N* = 3, see Fig. 4). The remaining twenty-nine patients (13 women, age 73 ± 9 years, BMI 26.7 ± 6.6 kg/m^2^) were included in the study. On average, 477 ± 98 mg/kg iodine was intravenously administered. Mean volume CT dose index and dose-length-product were 7.9 ± 2.3 mGy and 164 ± 55 mGy × cm, respectively. Applying a conversion factor of 0.014 mSv × mGy^−1^cm^−1^, the effective radiation exposure associated with LIE scans was determined at 2.95 ± 1 mSv (Table 2). Within the study sample, 19 patients displayed LIE patterns indicative of myocardial scarring (65.5%). No focal non-ischemic LIE pattern suggestive of myocarditis or non-ischemic cardiomyopathy was observed. Isolated hinge-point enhancement, when present, was considered a non-specific finding and was not classified as ischemic-type myocardial scar.

**Table 2:** Patient characteristics

Sex (women/men)	13/16
Age (years)	73 ± 9
Body mass index (kg/m^2^)	26.7 ± 6.6
Contrast volume (mL)	93 ± 17.6
Iodine dose (mg/kg)	477 ± 98
Volume CT dose index (mGy)	7.9 ± 2.3
Dose-length product (mGy × cm)	164 ± 55
Effective radiation dose (mSv)	2.95 ± 1
Late enhancement present (*n*)	19/29 (65.5%)

Continuous variables are given as mean ± standard deviation. For estimation of the effective radiation dose, a conversion factor of 0.014 mSv * mGy^−1^cm^−1^ was applied

### Objective image quality

Five minutes after contrast administration, iodine washout of healthy myocardial tissue was almost complete with low residual attenuation of 25.97 ± 8.7 HU. Contrarily, attenuation in the blood pool was still markedly increased at 58.8 ± 15.1 HU. Iodine retention in late-enhancing scar tissue resulted in a high signal attenuation of 53.5 ± 10.8 HU, comparable to blood pool (*p* = 0.079). Relative contrast enhancement between myocardium and blood pool was 34.3 ± 6.6 HU for all patients. In the subgroup of patients with LIE, relative enhancement between myocardium and the blood pool was higher than between myocardium and scar (34.3 ± 6.6 HU *versus* 26.5 ± 9.8 HU, absolute difference 7.8 ± 10.3 HU, *p* = 0.049). Image noise was generally very low (1.9 ± 0.6 HU), resulting in CNR for healthy myocardium (*versus* blood pool) of 20.0 ± 8.0. In the subgroup of patients with LIE, CNR of myocardium *versus* blood pool was higher than CNR *versus* late enhancing lesions (21.2 ± 7.8 *versus* 16.2 ± 7.3, *p* = 0.046). Table 3 provides an overview of quantitative imaging parameters.

**Table 3:** Quantitative image quality parameters

Contrast-to-noise ratios	
Myocardium *versus* blood pool (all)	20.0 ± 8.0
Myocardium *versus* blood pool (pat. with LIE)	21.2 ± 7.8
Myocardium *versus* late-enhancing lesions	16.2 ± 7.3
Relative contrast enhancement [HU]	
Myocardium *versus* blood pool (all)	33.9 ± 9.8
Myocardium *versus* blood pool (pat. with LIE)	34.3 ± 6.6
Myocardium *versus* late-enhancing lesion	26.5 ± 9.8

Continuous variables are given as mean ± standard deviation

### Diagnostic confidence

**Fig. 5:**
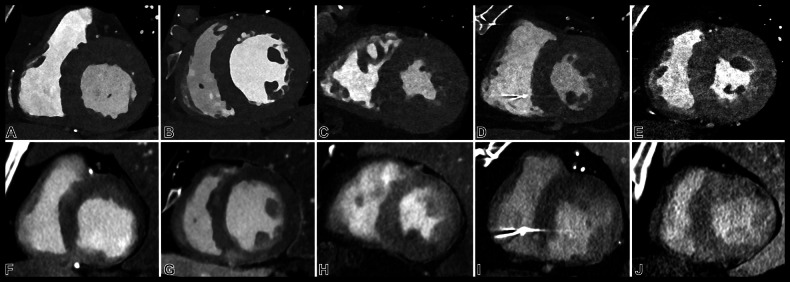
Image quality range of iodine maps for late enhancement. Exemplary images from the study cohort of 29 patients. The top row depicts short-axis reconstructions from coronary CT angiography (**A**-**E**), while the bottom row depicts corresponding iodine maps acquired 5 min after contrast injection (**F**-**J**). Image pairs **A/F, B/G, C/H, D/I** and **E/J** are sorted by weighted sum ranks of diagnostic confidence from best (**A**, **F**) to worst (**E**, **J**). Notice the gradual scarring inferior and inferolateral in **F**, hinge point fibrosis in **G**, and inferoseptal transmural scarring in **I**. The figure illustrates the range of image quality observed in this feasibility cohort, including cases in which subtle enhancement was difficult to assess while transmural enhancement remained visually appreciable

There were no non-diagnostic scans. The median diagnostic confidence to assess or interpret LIE in 25% of myocardium thickness was moderate (median 3, IQR 3–4), good up to 50% scarring (median 2, IQR 1–3) and high for assessing more than 50% tissue involvement (median 1, IQR 1-2). Diagnostic confidence increased substantially with higher LIE extent (*p* < 0.001). Further details can be found in Table 4 with corresponding imaging examples illustrating the spectrum of image quality achievable with the proposed protocol provided in Fig. 5.

**Table 4:** Readers’ confidence to interpret ischemic type late iodine enhancement

	- 25% myocardium thickness		- 50% myocardium thickness		> 50% myocardium thickness		overall image quality	
Confidence	absolute	relative	absolute	relative	absolute	relative	absolute	relative
Poor (5)	38	16.4%	11	4.7%	2	0.9%	19	8.2%
Fair (4)	62	26.7%	22	9.8%	7	3.0%	30	12.9%
Moderate (3)	78	33.6%	47	20.3%	29	12.5%	45	19.4%
Good (2)	36	15.5%	79	34.1%	57	24.6%	74	31.9%
High (1)	18	7.8%	73	31.5%	137	59.1%	64	27.6%
Median (IQR)	3 (3–4)		2 (1–3)		1 (1–2)		2 (1–3)	
ICC (significance)	0.795 (*p* < 0.001)		0.841 (*p* < 0.001)		0.834 (*p* < 0.001)		0.892 (*p* < 0.001)	

Shown are confidence ratings pooled from eight readers in 29 patients, resulting in 232 ratings per class, whereas absolute values indicate the frequency of the respective rating. Confidence was significantly different between classes (*p* < 0.001)

## Discussion

This retrospective feasibility study showed that PCD-CT-derived iodine maps acquired 5 min after coronary CTA without additional contrast administration provided measurable scar–myocardium contrast and high reader confidence for evaluating transmural LIE exceeding 50% myocardial wall thickness in a selected cohort. Reader confidence was lower for subtle subendocardial enhancement. These findings support further investigation of PCD-CT-derived iodine maps as a potential adjunct in selected patients in whom myocardial scar information is clinically relevant during CT work-up, but they do not establish diagnostic accuracy, clinical outcome benefit or equivalence to LGE-MRI. Importantly, some factors that limit MRI, such as severe obesity, inability to lie flat, arrhythmia or renal impairment, may also affect CT image quality or contrast suitability.

Prior dual-energy CT studies have shown that spectral post-processing can support delayed myocardial enhancement assessment, but image noise and limited CNR remain relevant challenges. PCD-CT may offer technical advantages through improved dose efficiency, reduced electronic noise, and availability of spectral information in cardiac acquisition modes. In the present study, scar–myocardium CNR on iodine maps was 16.2 ± 7.3, and reader confidence was high for LIE exceeding 50% transmural extent. However, because no direct DECT or EID-CT comparison was performed, these findings should be interpreted as feasibility data rather than evidence of superiority.

Prior studies have reported agreement between CT-derived LIE and MRI-derived LGE, providing the diagnostic accuracy context for CT-based scar imaging [17, 24–28]. Recent research strengthened the evidence base for PCD-CT in LIE imaging. In particular, Chamberlin et al [24] compared PCD-CT with energy-integrating detector CT for LIE in a substantial cohort and included an MRI-reference subset. Therefore, the novelty of the present study should not be understood as establishing diagnostic accuracy or technical superiority of PCD-CT. Rather, our contribution is a focused feasibility and reader-confidence assessment of PCD-CT-derived iodine maps acquired after coronary CTA without additional contrast administration, with confidence ratings stratified by transmural extent. In addition, the present study did not include a systematic reference standard and therefore cannot independently confirm such agreement. Accordingly, our results should be interpreted as image-quality and reader-confidence data only.

However, this study is the first to investigate the ubiquitous spectral post-processing capabilities of PCD-CT by focusing on the image quality and diagnostic benefits of dedicated iodine maps in the detection of ischemic LIE patterns. Considering substantial agreement between CT-derived LIE and MRI-derived LGE in identifying clinically significant scarring exceeding 50% of myocardial wall thickness [29], which is commonly used as a marker of limited likelihood of functional recovery after revascularization, our results encourage integrating LIE in standard cardiac CT workflows and conducting larger studies confirming the implications drawn from our results. However, conversely, lower confidence for enhancement involving ≤ 25% wall thickness indicates that subtle subendocardial scar remains challenging and requires dedicated validation against LGE-MRI.

A particular challenge of LIE imaging lies in the low inherent contrast due to substantially delayed image acquisition. While this issue is partly addressed by spectral post-processing techniques, such as virtual monoenergetic imaging with low-keV settings or iodine maps, these methods are associated with higher image noise ratios [30, 31]. To counteract the noise increase and to improve CNR at the myocardial interface, various approaches were employed in this study, such as the use of dedicated soft tissue kernels, higher slice thickness, and maximum iterative reconstruction strength, in addition to image acquisition in the end-systolic phase to increase detectability of significant scarring in the thickened myocardium. While the sum of these approaches led to good diagnostic confidence in the present investigation, the individual influence of each factor was not evaluated. Further improvements could be made by using advanced denoising techniques, such as deep learning-based algorithms [32].

Notably, the selection of reconstruction settings led to blurred junctions between the contracted trabecular and dense myocardium. The accompanying coronary CTA dataset was available as an anatomical reference for myocardial borders and may have reduced uncertainty regarding the actual myocardial boundaries when assessing the extent of LIE. However, the incremental effect of this reference on reader confidence was not formally evaluated. Our findings indicate that the image quality of iodine quantification maps is sufficient to detect clinically significant scarring with high confidence, whereas readers were less confident when assessing LIE patterns involving less than 25% of the myocardial thickness. The clinical implications of this result remain to be determined.

The radiation dose applied for LIE scans in this investigation was approximately 8 mGy (CTDI_Vol_) or 3 mSv effective dose, which is lower compared to earlier studies using energy-integrating detector systems, *e.g*., by Liu et al (15.0 ± 4.5 mGy) [17] and Oda et al (22.2 ± 5.5 mGy) [27]. Mergen et al realized an even greater effective dose reduction (1.2 mSv) [33] with a first-generation PCD-CT system, but the authors did not provide comparable image quality data. Consequently, further studies are needed to determine the optimal radiation dose for LIE imaging following the ALARA principle (“as low as reasonably achievable”).

With an iodine dosage of 477 ± 98 mg/kg, our study is comparable to other investigations applying between 300 and 700 mg of iodine per kg bodyweight [34]. LGE in MRI excels with high contrast of scarring against healthy myocardium, but also against the blood pool. Whereas the CNR of healthy myocardium (*versus* the blood pool) was high as well with our PCD-CT scan protocol, the contrast measured between scar and myocardium was slightly but significantly lower than that against the blood pool. Other authors have reported increased image contrast when using substantially higher iodine doses [25]; however, in light of our findings, a further increase in contrast volume might not be justifiable. Another factor that influences image contrast in the late phase is the delay between contrast agent administration and image acquisition. While literature recommends acquiring LIE scans 5 min after the iodine bolus for optimal contrast [35, 36], other settings may yield different results.

Since MRI capacities will presumably not significantly increase in the near future, the expected market penetration of PCD-CT may help radiologists to meet the increasing demands for myocardial viability assessment resulting from current guideline recommendations [37]. In this context, retrospective acquisition coronary CTA, which also provides functional data and LIE information, appears especially promising as a diagnostic “one-stop-shop” tool. While some authors even include first-pass perfusion to further complement the imaging workup [26], LIE may be the more important addition to cardiac CT protocols in patients with advanced ischemic cardiomyopathy or before complex percutaneous coronary interventions and bypass graft surgery.

The present study should be regarded as a technical proof-of-concept and workflow feasibility analysis. Its results may inform future prospective validation studies by suggesting that transmural LIE is assessable with high confidence, whereas subtle subendocardial enhancement requires particular attention. In the long term, delayed iodine maps could contribute to more comprehensive cardiac CT protocols that combine coronary anatomy with selected myocardial tissue information. In the present implementation, however, LIE imaging required an additional delayed acquisition and should therefore be considered a targeted add-on for selected patients rather than a routine one-stop-shop protocol. Future studies should include larger multicenter cohorts, systematic LGE-MRI reference, predefined segment-based analysis, assessment of clinical impact, and comparison with conventional delayed CT reconstructions

Several limitations must be acknowledged for this study. First, the small sample size limits the generalizability of the reported results. Therefore, the results should be considered hypothesis-generating and should not be generalized to unselected coronary CTA populations. Second, CT-derived LIE was not intra-individually compared to MRI-derived LGE, since MRI examinations were not available in most patients and illustrative cases with prior MRI are useful for anatomical orientation, but they cannot replace systematic intra-individual validation. Accordingly, the present work does not address diagnostic accuracy, sensitivity, specificity or agreement with CMR, and conclusions are limited to image quality, scar conspicuity and reader confidence. At the same time, the cohort reflects a selected real-world CT workflow in which LIE was performed when additional myocardial scar information was considered clinically relevant during coronary CTA work-up. In addition, no matched conventional delayed enhancement CT, energy-integrating detector CT, or dual-energy CT acquisition was available. Consequently, the present study cannot determine whether PCD-CT iodine maps are superior to conventional delayed CT techniques. However, very good correspondence between CT LIE and MRI LGE was demonstrated by others, and there is no reason to believe that using iodine maps for LIE assessment might diminish comparability. Third, the clinical impact of LIE findings was not assessed. In light of our results, which encourage employing LIE in broader cohorts, this will be a major topic for further studies. Fourth, no conclusions about other entities of myocardial damage, such as peri-/myocarditis or diffuse fibrosis, can be drawn from this study. Fifth, the quantitative nature of iodine maps and their potential to determine the extracellular volume, which may further enhance the diagnostic value of PCD-CT-derived LIE, was not investigated. Sixth, readers’ confidence ratings were not calibrated. CT LIE imaging was not a standard modality in the reader's institution previously to this work, but all readers were conversant with LGE interpretation. This low level of experience with CT LIE might have negatively impacted confidence ratings, with resulting understimation of subjective image quality. Seventh, while the multi-reader design is a strength of this feasibility study and good inter-reader agreement was reached despite variable cardiac imaging experience, the study was not designed or powered to compare experience subgroups and no calibrated training session was performed. Future studies should use standardized reading instructions, training cases, and segment-based scoring to assess whether reader experience influences diagnostic confidence or accuracy. Finally, the selection mechanism, which excluded patients without apparent CAD, may lead to overestimating the feasibility or confidence in routine unselected populations.

## Conclusion

PCD-CT-derived iodine maps acquired 5min  after coronary CTA without additional contrast administration provided measurable scar–myocardium contrast and high reader confidence for evaluating transmural LIE exceeding 50% myocardial wall thickness in this small, selected feasibility cohort. Confidence was lower for subtle subendocardial enhancement. Prospective studies with systematic LGE-MRI reference are required to determine diagnostic accuracy, clinical impact, and the role of this approach in cardiac CT workflows.

## Data Availability

The datasets generated and/or analyzed during the current study are not publicly available but are available from the corresponding author on reasonable request. Due to the nature of this research, participants of this study did not agree for their data to be shared in a public repository.

## Abbreviations

CNR: Contrast-to-noise ratio
CTA: Computed tomography angiography
LIE: Late iodine enhancement
LGE: Late gadolinium enhancement
PCD-CT: Photon-counting detector computed tomography
